# Supplementary material for: Empirical methods for controlling false positives and estimating confidence in ChIP-Seq peaks
Source: BMC Bioinformatics. 2008 Dec 5;9:523. doi: 10.1186/1471-2105-9-523 (PMC2628906; doi:10.1186/1471-2105-9-523)
Supplement: Additional File 1 — A variety of html documents from the USeq web site detailing the available applications, their best usage, output file type descriptions, command line menus, etc. [file 1471-2105-9-523-S1.zip › USeqUserGuides/JavaDoc.zip!/JavaDoc/bioroot/Gene.html]

Gene


|  |  |  |
| --- | --- | --- |
| **Overview** \| **Package** \| **Class** \| **Use** \| **Tree** \| **Deprecated** \| **Index** \| **Help** | |  |
| **PREV CLASS**   **NEXT CLASS** | **FRAMES**    **NO FRAMES**     **All Classes** |  |
| SUMMARY: NESTED \| FIELD \| CONSTR \| METHOD | DETAIL: FIELD \| CONSTR \| METHOD |  |


---


## bioroot Class Gene

```
java.lang.Object
  javax.servlet.GenericServlet
      javax.servlet.http.HttpServlet
          bioroot.Gene
```

**All Implemented Interfaces:**: java.io.Serializable, javax.servlet.Servlet, javax.servlet.ServletConfig

---

``` public class Gene extends javax.servlet.http.HttpServlet ```

**Author:**
:   nix

**See Also:**: Serialized Form

---

**Constructor Summary**

`Gene()`


| **Method Summary** | |
| --- | --- |
| `void` | `doGet(javax.servlet.http.HttpServletRequest request, javax.servlet.http.HttpServletResponse response)` |
| `void` | `doPost(javax.servlet.http.HttpServletRequest request, javax.servlet.http.HttpServletResponse response)` |

**Methods inherited from class javax.servlet.http.HttpServlet**

`service`

**Methods inherited from class javax.servlet.GenericServlet**

`destroy, getInitParameter, getInitParameterNames, getServletConfig, getServletContext, getServletInfo, getServletName, init, init, log, log`

**Methods inherited from class java.lang.Object**

`equals, getClass, hashCode, notify, notifyAll, toString, wait, wait, wait`

**Constructor Detail**

### Gene

```
public Gene()
```


**Method Detail**

### doGet

```
public void doGet(javax.servlet.http.HttpServletRequest request,
                  javax.servlet.http.HttpServletResponse response)
           throws javax.servlet.ServletException,
                  java.io.IOException
```

:   **Overrides:**: `doGet` in class `javax.servlet.http.HttpServlet`
:   **Throws:**: `javax.servlet.ServletException`: `java.io.IOException`

---


### doPost

```
public void doPost(javax.servlet.http.HttpServletRequest request,
                   javax.servlet.http.HttpServletResponse response)
            throws javax.servlet.ServletException,
                   java.io.IOException
```

:   **Overrides:**: `doPost` in class `javax.servlet.http.HttpServlet`
:   **Throws:**: `javax.servlet.ServletException`: `java.io.IOException`


---


|  |  |  |
| --- | --- | --- |
| **Overview** \| **Package** \| **Class** \| **Use** \| **Tree** \| **Deprecated** \| **Index** \| **Help** | |  |
| **PREV CLASS**   **NEXT CLASS** | **FRAMES**    **NO FRAMES**     **All Classes** |  |
| SUMMARY: NESTED \| FIELD \| CONSTR \| METHOD | DETAIL: FIELD \| CONSTR \| METHOD |  |


---
